# Severity of psychotic episodes in predicting concurrent depressive and anxiety features in acute phase schizophrenia

**DOI:** 10.1186/1471-244X-14-166

**Published:** 2014-06-05

**Authors:** Kalai Naidu, Werdie (CW) van Staden, Mike van der Linde

**Affiliations:** 1 Department of Psychiatry, University of Pretoria, Private Bag X323, Arcadia, Pretoria 0007, South Africa; 2 Department of Statistics, University of Pretoria, Pretoria, South Africa

**Keywords:** Acute psychosis, Severity, Akathisia, Symptomatology, Symptoms

## Abstract

**Background:**

Considering that depressive and anxiety symptoms are common in schizophrenia, this study investigated whether the severity of a psychotic episode in an acute phase schizophrenia cohort is predictive of concurrent depressive and anxiety features.

**Method:**

Fifty one recently hospitalised patients suffering from acute phase schizophrenia participated prospectively in a cross-sectional study. The severity of the psychotic episode, the depressive features and the anxiety features were measured by the Structured Clinical Interview for Positive and Negative Syndrome Scale (SCI-PANSS), the Calgary Depression Scale for Schizophrenia (CDSS), the Hamilton Anxiety Rating Scale (HAM-A) and the Staden Schizophrenia Anxiety Rating Scale (S-SARS). The total SCI-PANSS-scores were adjusted to exclude appropriately the depression or anxiety items contained therein. To examine akathisia as potential confounder, the Barnes Akathisia Scale was also applied. The relationships were examined using linear regressions and paired t-tests were performed between lower and higher scores on the SCI-PANSS.

**Results:**

A higher adjusted total SCI-PANSS-score predicted statistically significantly higher scores for depressive features on the CDSS (p < 0.0001) and for anxiety features on the HAM-A (p = 0.05) and the S-SARS (p < 0.0001). The group that scored more or equal to the median (=99) of the adjusted total SCI-PANSS, scored significantly higher (p < 0.0001) on the CDSS, the HAM-A and the S-SARS than the group scoring below it. Akathisia measured distinctly different (p < 0.0001) from both the anxiety measures.

**Conclusion:**

The study suggests that the severity of a psychotic episode in acute phase schizophrenia predicts the severity of concurrent depressive and anxiety features respectively.

## Background

Although not defining features of schizophrenia, depressive and anxiety features present commonly in patients suffering from schizophrenia [1]. The lifetime prevalence of depression in patients with schizophrenia is reported to be 60 to 80% [2-5]. This suggests a significant risk when compared with a lifetime prevalence of depression of 8–26% in the general population [6]. For anxiety disorders, a meta-analysis by Achim et al. found a pooled prevalence rate of 38.3% in schizophrenia with a 95%-confidence interval of 26.3% to 50.4%, which seems also higher than the 28.8% reported for the general population [7,8].

A rather complex relationship between schizophrenia symptoms and features of depression had already been the interest of Bleuler, among others, at the beginning of the 20^th^ century. Bleuler described the relationship as follows: “the special position of depressive mood swings in schizophrenia varies from case to case and in some cases seems to be triggered by the schizophrenic disease process, and in others takes the role of secondary symptoms…” [9]. Congruently, another study found differential prevalence rates of 52% in acutely psychotic patients and 38% during more stable periods of the disease [10].

The notion that depressive features in schizophrenia are limited to the post-psychotic period has been challenged by many studies that have established that depressive features are also present during the acute psychotic phase of the illness [10-12]. Hirsch et al. suggest that the highest rate of depression in patients with schizophrenia is found during the acute episode [13]. At time of admission to hospital, half of the acutely ill psychotic patients with schizophrenia present with significant depressive features. Some of these patients experience a spontaneous remission of the features within 3 weeks, while about half develop persistent depressive features. Another study found a remission rate of even 98% as the psychosis remitted [12].

Concurrent depressive features in schizophrenia patients are associated with increased morbidity and mortality [14]. In this cohort study of 7217 patients in Finland conducted over a 17 year period, it was shown that accompanying depressive features in schizophrenia incur elevated risks for both natural and unnatural mortality [14]. Suicide was one of the leading causes of premature death among these patients, and was related to the severity of depressive features. Also in another study, depressive features accounted for nearly 50% of the suicidal intent seen in a sample of 267 patients between the ages of 18 and 70 [15].

Depressive features are associated, furthermore, with an increased rate of relapse, more frequent and longer duration of hospital admissions and a poor response to pharmacological treatments [2]. Cognitive impairment, substance abuse, poor social functioning, and suicide or suicide attempts have also been reported [5,16]. Neuropsychological impairments seen in schizophrenia, especially of memory and attention, have also been linked with depressive features [16]. The relationship between schizophrenia and anxiety features is also complex as suggested by the variable prevalence figures for the respective anxiety disorders [7]. Statistically significant heterogeneity among prevalence figures was found, spanning all kinds of anxiety disorders. As with the severity of depressive features, the severity of anxiety is also of clinical significance. Anxiety disorders on their own can adversely affect quality of life, mobility, education, employment, social functioning, health care, and physical well-being [17]. Patients with both schizophrenia and anxiety features suffer from more global functional impairment as well as more severe limitations in the domains of employment and social life – suggesting that anxiety features in these patients bring about an additional burden to their already compromised quality of life [18].

Social anxiety disorder in schizophrenia is associated with a higher lifetime rate of suicide attempts, greater lethality of suicide attempts, more past substance/alcohol abuse disorder, lower social adjustment, and overall quality of life [19]. In a study by Mazeh et al., which investigated the prevalence and correlates of social anxiety disorder in patients with schizophrenia, significant correlation was found between the score of the Liebowitz Social Anxiety Scale and the positive symptom subscale of the PANSS, which highlights the complex interaction between anxiety and schizophrenia [20]. Comorbid panic attacks in schizophrenia are associated with depressive features and have a negative impact on quality of life when compared to healthy controls in all domains of the World Health Organization Quality of Life Instrument [21]. Also in a longitudinal study, anxiety disorders were found to be the best predictor of subjective quality of life in patients with schizophrenia [22].

The relationship of schizophrenia with depressive and anxiety features respectively needs to be investigated further, considering the clinical importance and prevalence of these in schizophrenia. Considering that this relationship may be different in the acute psychotic phase compared to the post-psychotic phase, this study investigated a cohort of patients during the acute phase of schizophrenia for concurrent depressive and anxiety features. More specifically, it enquired for the first time as far as we could establish, whether the severity of a psychotic episode is predictive of concurrent depressive and anxiety features among recently hospitalised patients in acute phase schizophrenia.

## Methods

### Participants

Fifty one patients between the ages of 18 to 65 who were suffering from an acute phase of schizophrenia were prospectively included within ten days of their admission to the Weskoppies Psychiatric Hospital in Pretoria, South Africa. For inclusion, they met the DSM-IV-TR criteria for schizophrenia [23], and the acute phase was verified by a minimum total score requirement of 60 on the Structured Clinical Interview for Positive and Negative Syndrome Scale (SCI-PANSS) as well as a score of at least 4 on any 2 of the SCI-PANSS items that constitute a psychotic item subscale (these are, hallucinatory behaviour, delusions, conceptual disorganisation, and suspiciousness).

Patients were excluded if they suffered from another psychiatric diagnosis in addition to schizophrenia; a neurological condition affecting the central nervous system; substance dependence; or who were likely to suffer from substance withdrawal symptoms. Patients with schizoaffective disorder were excluded by applying the C-criterion of DSM-IV diagnostic criteria for schizoaffective disorder, viz. that symptoms that meet criteria for a mood episode are present for a substantial portion of the total duration of the active and residual periods of the illness. In addition, patients with a history of taking benzodiazepines regularly during the past month were excluded as well as patients who received zuclo-penthixol acetate less than 72 hours prior to applying the measures of this study.

### Measures

The Structured Clinical Interview for Positive and Negative Syndrome Scale (SCI-PANSS) was used to measure the severity of the psychotic episode [24]. Since this scale contains specifically an item for depression and one for anxiety, each of these were excluded from the total SCI-PANSS score and are reported here as two adjusted total scores (that is, a total SCI-PANSS score minus respectively the depression and anxiety items).

Depressive features were measured by the Calgary Depression Scale for Schizophrenia that was developed specifically for schizophrenia patients [25]. It is an observer rated scale that consists of 9 items, each with 4 anchor points. Anxiety was measured by two observer rated scales: the Hamilton Anxiety Rating Scale (HAM-A), which has 14 items, each of which is rated from 0 (none) to 4 (severe, grossly disabling) [26]. It is not specifically designed for the schizophrenia population and marks mostly bodily and behavioural manifestations of anxiety; In addition, the Staden Schizophrenia Anxiety Rating Scale (S-SARS) was used, consisting of 10 items, each with 6 narrative anchor points descriptive of severity. The items are persecutory and nihilistic anxiety; perceptual anxiety; anxiety attacks; situational anxiety; obsessive-compulsive anxiety; somatic anxiety; psychomotor and cognitive anxiety; worry and fear; control-related anxiety, and impairment from anxiety.

To distinguish anxiety from akathisia, an extrapyramidal side effect of commonly used medication for this group of patients [11], the Barnes Akathisia Scale was used. It is an observer rated scale consisting of 4 items with 6 anchor points each [27].

Descriptive variables included age, gender, medication used during the past week, depot medication during the past month, and the number of previous hospital admissions.

### Statistical analyses

Means and standard deviations on the total scores (continuous variables) obtained in the administration of the respective measurement instruments were calculated. Statistical testing was done by means of linear regression. As an alternative statistical strategy, the median of the adjusted total SCI-PANSS-scores was used to demarcate lower and higher scores, on which paired t-tests (upper sided) were performed. The paired t-test (two-sided) was also performed between the measure of akathisia and the respective anxiety measures.

### Ethical provisions

The Faculty of Health Sciences Research Ethics Committee of the University of Pretoria provided ethics approval for the study and the hospital’s chief executive officer provided the necessary permission. All participants gave written informed consent after they had been clinically assessed as being capable of doing so [28,29]. Patients who were not capable of consenting were excluded from the study. The study was executed in compliance with the Declaration of Helsinki (2008).

## Results

### Demographic, hospitalisation and medication characteristics of participants

Fifty one patients were recruited in this study with a mean age of 37.02 years (SD = 13.29). Thirty nine subjects were male (76.47%) and 12 were female (23.53%). The mean number of previous admissions was 4.06 (SD = 2.55). All of the participants had been hospitalised at psychiatric units previously. Seven had been admitted once before (13.73%), and 13 had been admitted at least 3 times before (25.49%). Twenty nine patients (56.86%) used one oral antipsychotic medication, whereas 6 (11.76%) used none. A third of patients (n = 17) were on a depot antipsychotic medication.

### Symptom characteristics of participants

Participants experienced severe psychotic features as suggested by a mean total SCI-PANSS score of 97.61 (SD = 17.83), ranging from 60 to 146. The mean on the positive symptoms subscale was 26.18 (SD = 4.87), ranging between 10 and 34. The negative symptoms subscale scored a mean of 24.2 (SD = 5.8), ranging between 12 and 39. The general psychopathology subscale scores ranged between 26 and 77 with a mean of 47.12 (SD = 10.84).

Regarding depressive features, 7.6% of participants scored zero on all items of the Calgary Depression Scale for Schizophrenia. The mean total score was 6.45 (SD = 5.50; ranging to 21). Thirteen of the participants had suicidal ideation of which 6 (11.8%) were considered to be severely suicidal, having scored maximum on the suicide rating item of the scale.

Regarding anxiety features, 3 participants (5.88%) scored zero on all items of the Hamilton Anxiety scale. The mean score was 11.29 (SD = 6.68), ranging from one to 27. The scores on the S-SARS ranged from zero to 42, with a mean of 18.88 (SD = 10.13). The specific anxiety subscale, with items on persecutory and nihilistic anxiety, perceptual anxiety, anxiety attacks, situational anxiety and obsessive-compulsive anxiety, yielded a mean of 8.80 (SD = 5.51; range 0 – 23). The general anxiety subscale, with items on somatic anxiety, psychomotor and cognitive agitation, worry and fear, control-related anxiety and impairment from anxiety yielded a mean of 10.08 (SD = 5.12; range 0 – 19).

Eighteen participants (35.3%) had no features of akathisia according to the Barnes Akathisia Scale. The mean score was low at 1.67 (SD = 1.96) ranging from 0 to 9.

### Relationships between severity of psychotic features and, respectively, depressive and anxiety features

A higher adjusted total SCI-PANSS-score (i.e., the total SCI-PANSS-score minus the depression item) predicted statistically highly significantly a higher score on the Calgary Depression Scale for Schizophrenia (CDSS) (see linear regression results in Table 1). Moreover, the group that scored more or equal to the median (with the median being 99) of the total SCI-PANSS, scored significantly higher on the CDSS than the group below this median (see the paired t-test figures in Table 1).

**Table 1:** Total SCI-PANSS predicting CDSS, HAM-A-and S-SARS

**Measures**		**Linear regression**		**Paired t-tests (upper-sided)**			
		**F-value**	** p-value**	**Difference of means**	**Confidence Interval**	**t-value**	** p-value**
SCI-PANSS without depression item	**CDSS**	27.73	< 0.0001**	88.55	87.26 to infinity	114.97	< 0.0001**
SCI-PANSS without anxiety item	**HAM-A**	4.18	0.05*	83.71	82.14 to infinity	89.50	< 0.0001**
SCI-PANSS without anxiety item	**S-SARS (total)**	31.05	< 0.0001**	76.12	73.74 to infinity	53.68	< 0.0001**
SCI-PANSS without anxiety item	**General Anxiety Subscale of S-SARS**	29.14	< 0.0001**	84.92	83.72 to infinity	118.50	< 0.0001**
SCI-PANSS without anxiety item	**Specific Anxiety Subscale of S-SARS**	24.51	< 0.0001**	86.20	84.90 to infinity	111.72	< 0.0001**

*= statistically significant. **= statistically highly significant.

Similarly for anxiety: the linear regression analyses yielded statistically significant results for the adjusted total SCI-PANSS-score (i.e., total SCI-PANSS-score minus the anxiety item) predicting higher scores on, respectively, the Hamilton Anxiety Scale (HAM-A) and the Staden Schizophrenia Anxiety Rating Scale (S-SARS). Comparing the group that scored higher or equal to the median of the total SCI-PANSS-score to the lower scoring group, yielded statistically highly significant predictions of higher scores on the HAM-A and the S-SARS (see Table 1). Statistically highly significant results were also found for the subscales of the S-SARS.

To explore more about the details contained within the total scores, the sub-scores were also examined albeit rather tentatively considering the modest sample size. For the SCI-PANSS-subscales, viz. the Positive Syndrome Subscale; the Negative Syndrome Subscale; and the General Psychopathology Subscale, linear regressions were performed to ascertain the contributions of the SCI-PANSS-subscales to the predicative relationships with respectively the CDSS, the HAM-A, the S-SARS and the subscales of the S-SARS. The results are presented in Table 2, showing that higher scores on respectively the General Psychopathology Subscale (as appropriately adjusted) and the Negative Syndrome Subscale predicted higher values (statistically highly significantly) for the CDSS, S-SARS and the subscales of the S-SARS. However, a similar relationship between the scores of the Negative Syndrome Subscale and the HAM-A did not emerge. Results did not support scores of the Positive Syndrome Subscale predicting the scores of the CDSS, but approached statistical significance for predicting the scores on the measures of anxiety (except for the General Anxiety Subscale of the S-SARS that yielded statistically significant values).

**Table 2:** Subscales of the SCI-PANSS predicting CDSS, HAM-A and S-SARS

**Measures**	**Linear Regression**	
	**F-value**	**p-value**
**SCI-PANSS Positive Syndrome Subscale**		
** CDSS**	7.93	0.39
** HAM-A**	3.73	0.06
** Total S-SARS**	3.52	0.07
** S-SARS General Anxiety Subscale**	4.28	0.04*
** S-SARS Specific Anxiety Subscale**	2.24	0.14
**SCI-PANSS Negative Syndrome Subscale**		
** CDSS**	23.64	< 0.0001**
	** HAM-A**	0.84	0.44
	** Total S-SARS**	11.61	0.001**
	** S-SARS General Anxiety Subscale**	11.34	0.001**
	** S-SARS Specific Anxiety Subscale**	9.43	0.004**
**SCI-PANSS General Psychopathology Subscale minus depression item**			
** CDSS**	27.02	< 0.0001**	
**SCI-PANSS General Psychopathology Subscale minus anxiety item**			
** HAM-A**	4.07	0.05*	
** Total S-SARS**	37.13	< 0.0001**	
** S-SARS General Anxiety Subscale**	31.83	< 0.0001**	
** S-SARS Specific Anxiety Subscale**	31.20	< 0.0001**	

*= statistically significant. **= statistically highly significant.

The concern that akathisia as an adverse effect of antipsychotic medication may appear like anxiety in examining the relationships as reported above, was not sustained by the results. The measure of akathisia measured distinctly from the anxiety measures. Statistically highly significant differences between the Barnes Akathisia Scale and the measures of anxiety were found: these are, for the HAM-A (mean difference = 9.63; 95% CI = 7.76 to 11.49; t = 10.37; p < 0.0001) and for the S-SARS (mean difference = 17.22; 95% CI = 14.54 to 19.89; t = 12.92; p < 0.0001). The total Barnes scores neither proved to be a statistically significant covariant to the adjusted total SCI-PANSS predicting respectively the HAM-A (F = 0.01; p = 0.93) and S-SARS (F = 0.07; p = 0.8).

The measurements of depressive features on the CDSS in this cohort were found to be statistically highly significantly different from anxiety features as measured by the HAM-A (mean difference = 4.84; 95% CI = 3.0 to 6.69; t = 5.27; p < 0.0001) and the S-SARS (mean difference = 12.43; 95% CI = 10.24 to 14.63; t = 11.37; p < 0.0001). Similarly, the measurements on the adjusted SCI-PANSS were found to be statistically highly significantly different from the CDSS (mean difference = 87.88; 95% CI = 83.88 to 91.88; t = 44.12; p < 0.0001), the HAM-A (mean difference = 83.0; 95% CI = 78.35 to 87.65; t = 35.88; p < 0.0001), and the S-SARS (mean difference = 75.41; 95% CI = 71.65 to 79.18; t = 40.23; p < 0.0001).

## Discussion

The main finding of this study is that the severity of a psychotic episode in acute phase schizophrenia predicts the severity of concurrent depressive and anxiety features respectively. Whereas most of the previous studies examined prevalence figures for depressive and anxiety disorders in schizophrenia, this study reports these findings as far as we could establish for the first time in a cohort that suffered on average from a rather severe degree of psychotic episode. This study, moreover, measured anxiety in its various presentations without assuming that anxiety in acute phase schizophrenia necessarily present in the form of established diagnostic categories of anxiety disorders.

The results statistically differentiated anxiety from akathisia in this cohort, suggesting that akathisia is not a confounder in the finding that more severe anxiety is predicted by how severe the psychotic episode is. The finding that the severity of a psychotic episode in acute phase schizophrenia predicts the severity of concurrent depressive and anxiety features is further supported by the verification that the severity measurements in this study of the psychotic episode, the depressive features and the anxiety features are distinct. That indicates that the main finding of this study is not an artefact of overlap between the respective measure instruments. That also suggests that the instruments validly distinguished between clusters of respectively psychotic, depressive and anxiety features, notwithstanding the practical difficulties in distinguishing between specific symptoms of these kinds reported elsewhere (e.g., intrusive and repetitive delusional ideas vs. obsessions in obsessive compulsive disorder; hallucinatory experiences vs. flashbacks in post-traumatic stress disorder; delusional ideas of reference vs. fear of being judge in social anxiety disorder) [7,30].

One should be cautious about generalising the findings, however. That lesser severity of a psychotic episode predicts lesser degrees of depression and anxiety in acute phase schizophrenia, may mistakenly be taken to imply that people with schizophrenia who are not currently psychotic would not have significant depression and anxiety. That would be mistaken for this study should not be generalised to non-acute phase schizophrenia. On the contrary, previous studies suggested significant depressive and anxiety features in schizophrenia following psychotic episodes [31-33].

To generalise, furthermore, the study should first be replicated at other sites, using a larger sample size. Other factors that may influence the relationships found in this study should be studied further. For example, our study involved only hospitalised patients, meaning that non-hospitalised patients should be investigated too and compared with hospitalised patients. Furthermore, our study excluded such potential influences as having another psychiatric diagnosis in addition to schizophrenia; a neurological condition affecting the central nervous system; substance dependence; patients with a history of taking benzodiazepines regularly during the past month; and patients who received zuclo-penthixol acetate less than 72 hours prior to applying the measures of this study.

According to the clinical history of the longitudinal course of the disorder available at the time of admission, a mood episode must not have been present for a substantial portion of the total duration of the active and residual periods of the illness – thus not meeting the C-criterion of schizoaffective disorder [23]. Nonetheless, it is possible that some patients in this cohort could turn out to be suffering from schizoaffective disorder, particularly where the history might not have been not sufficiently telling at the time of admission, or where the disorder was yet to unfold in a way that the C-criterion would be met at a point after this study.

The finding that the severity of the psychotic episode predicts the severity of anxiety should be qualified further. That is, the anxiety measured was not in terms of specific anxiety *disorders*, but in terms of general measures of anxiety features. For example, whether the severity of a psychotic episode would predict the severity of obsessions and compulsions in patients with both obsessive compulsive disorder and acute phase schizophrenia is not addressed here but a question for further research. Nonetheless, this study prompts the related question that has not yet been thoroughly researched: does anxiety in acute phase schizophrenia necessarily present in the form of established diagnostic categories of anxiety disorders, or may people suffer from significant anxiety symptoms in acute phase schizophrenia even when those symptoms do not clearly cluster into the established diagnostic categories of anxiety disorders.

Our study suggests that the heavier burden owing to a more severe degree of a psychotic episode is made even heavier by more severe depressive and anxiety features at the time. The justified clinical attention to the psychotic symptoms in severe degrees of acute phase schizophrenia and the undoubted clinical challenges that these symptoms pose, may overshadow if not obscure the presence and importance of the depressive and anxiety features. However, appreciating the burden that entails for a specific patient is very important in an empathetic therapeutic relationship with the patient. It is also clinically important for constructing personalised treatment plans that account for the added burden as well as the anticipated increased risks for suicide, self-harm, violence, treatment non-adherence, and the impulsive or destructive behaviours associated to it [34]. Research is required for establishing the extent of these increased risks as well as into efficacious ways to alleviate the compounded burden.

## Conclusions

This study suggests that the severity of a psychotic episode in acute phase schizophrenia predicts the severity of concurrent depressive and anxiety features respectively. These results did not appear to be confounded by akathisia, and prompt the question not yet researched thoroughly as to whether clinically significant anxiety features in acute phase schizophrenia necessarily cluster into the established diagnostic categories of anxiety disorders.

## Competing interests

The authors declare that they have no competing interests.

## Authors’ contributions

KN & WVS conceptualised and designed the study, obtained research ethics approval, obtained consent from research participants, conducted the interviews and administered the measuring instruments, analysed and interpreted the data, and wrote the paper. MvdL provided statistical and data processing advice in the conceptualisation and design of the study, and performed the statistical analysis. All authors read and approved the final manuscript.

## Pre-publication history

The pre-publication history for this paper can be accessed here:

http://www.biomedcentral.com/1471-244X/14/166/prepub
